# Parkinson's Disease Treatment: A Bibliometric Analysis

**DOI:** 10.7759/cureus.69613

**Published:** 2024-09-17

**Authors:** Billy McBenedict, Wilhelmina N Hauwanga, Gustavo Ienaco, Dulci Petrus, Syeda Sukaina Kazmi, Jonatha Machado Lima, Barakat B Onabanjo, Asaju Felix, Sujood Awadelseed, Shivadeva Selvamani, Phoh Wen Cher, Bruno Lima Pessôa

**Affiliations:** 1 Neurosurgery, Fluminense Federal University, Niterói, BRA; 2 Family Medicine, Faculty of Medicine, Federal University of the State of Rio de Janeiro, Rio de Janeiro, BRA; 3 Medicine, Fluminense Federal University, Niterói, BRA; 4 Family Health, Directorate of Special Programs, Ministry of Health and Social Services, Windhoek, NAM; 5 Medicine, Ziauddin University, Karachi, PAK; 6 General Practice, Dorset County Hospital, Dorchester, GBR; 7 Medicine, International Medical University, Kuala Lumpur, MYS; 8 Family Medicine, International Medical University, Kuala Lumpur, MYS

**Keywords:** bibliometric analysis, deep brain stimulation, neurodegenerative disorders, parkinson's disease, research trends

## Abstract

Parkinson's disease (PD) is a progressive neurodegenerative disorder marked by motor symptoms like bradykinesia, tremor, rigidity, and postural instability. Patients also experience non-motor symptoms that greatly affect their quality of life. The global prevalence of PD is increasing, especially among the elderly, necessitating effective treatment strategies. This review provides an overview of the current treatment modalities for PD, including pharmacological and surgical interventions, and employs a bibliometric analysis to evaluate the trends and impact of scientific research in this field. A comprehensive search of the Web of Science Core Collection (WoSCC) database was conducted on July 12, 2024, yielding 3,724 publications related to PD treatment. Bibliometric analysis was performed using Biblioshiny and VOSviewer to assess publication trends, impact, and collaborative networks. Metrics such as the number of publications, citations, h-index, and country/institutional contributions were analyzed to identify key areas of focus and influential research in PD treatment. The analysis revealed a significant increase in PD research output from 2000 onwards, peaking between 2011 and 2016. The United States led in research production, followed by China, Canada, and the United Kingdom. Key researchers included Lang AE, Okun MS, and Lozano AM, with the University of Toronto, University of California System, and Harvard University being the top contributing institutions. The study identified major trends in pharmacological treatments, such as dopamine replacement therapy and deep brain stimulation (DBS) as the most common surgical intervention. Bibliometric analysis highlighted significant international collaborations and identified influential studies shaping the current understanding and treatment of PD. This bibliometric analysis elucidated the trends and impacts of scientific contributions, emphasizing the prolific output from leading countries and institutions in relation to the treatment of Parkinson's disease. Take-home messages for the conclusion of our study are as follows: (1) this study found a substantial increase in Parkinson's disease (PD) research output from 2000 onwards, peaking around 2017-2018, (2) noted a decline in publication output post-2020, (3) the United States had the highest research output, followed by significant contributions from countries like China, Canada, and the United Kingdom, (4) international collaborations played a vital role in advancing PD research, (5) key researchers in the field were Lang AE, Okun MS, and Lozano AM, (6) and established institutions like the University of Toronto, Johns Hopkins University and Harvard University made substantial contributions to the field, emphasizing the role of leading academic centers in driving PD research.

## Introduction and background

Parkinson's disease (PD) is a progressive neurodegenerative disorder characterized by motor symptoms such as tremor, rigidity, bradykinesia, and postural instability, along with non-motor symptoms that significantly impact the quality of life [1]. It is the second most common neurodegenerative disease, with the highest incidence and prevalence in individuals aged ≥65 years, making it a significant public health burden among the elderly [1]. The clinical course of the disease includes motor symptoms like resting tremor and rigidity, as well as non-motor symptoms such as autonomic dysfunction, sleep disorders, cognitive deficits, and behavioral changes. Symptoms of prodromal PD, such as constipation and insomnia, often manifest years before the appearance of classic motor symptoms [2]. Understanding of PD mechanisms has advanced, highlighting intracellular aggregation of α-synuclein forming Lewy bodies and loss of dopaminergic neurons, initially in the substantia nigra and later more widespread as the disease progresses [3].

The rising global prevalence of Parkinson's disease underscores the need for enhanced public health strategies and interventions, particularly in rapidly developing countries. Zhu et al.’s research showed a significant rise in Parkinson's disease prevalence from 1980 to 2023, especially between 2004 and 2023, with a global all-age prevalence of 1.51 cases per 1000 people, higher in males [1]. This increase is influenced by factors like human development index (HDI) and sociodemographic index (SDI), with middle SDI countries seeing the highest rates since 2010 [1]. The prevalence has increased across all regions, age groups, and sexes, driven by higher incidence, improved medical care, and population ageing [1]. Regional disparities are partly due to varying levels of population ageing and exposure to environmental risk factors [1]. This highlights the need for better public health strategies to address the growing global burden of Parkinson's disease. As the global prevalence of PD continues to rise, there is an increasing demand for effective treatment strategies to manage and alleviate the symptoms of this debilitating condition.

Over the years, a variety of therapeutic interventions have been developed and refined, ranging from pharmacological approaches to advanced surgical techniques. Current treatment options for PD are plentiful, at least in comparison to other neurodegenerative diseases, and offer PD patients extended control of symptom severity as well as an improved quality of life [4]. Unfortunately, no treatment halts the pathological mechanisms that drive disease progression, with most treatments being focused on replacing or enhancing dopamine availability [4]. The golden standard in pharmacologic therapy is dopamine replacement therapy, mainly levodopa, used in synergy with dopamine receptor agonists, monoamine oxidase (MAO) inhibitors or catechol-O-methyltransferase (COMT) inhibitors [4]. The challenge that stems from this type of therapy is the delicate balance between the beneficial and harmful effects that can arise [5]. Levodopa use is often delayed because of drug-induced dyskinesias and wearing-off and on-off fluctuations. A meta-analysis demonstrated that the MAO-B inhibitor rasagiline is effective both as a monotherapy and in combination with other treatments, with a safety profile comparable to a placebo [6]. Pramipexole, a nonergoline D3-preferring dopamine agonist, is also used to manage motor symptoms in PD [7].

Among the surgical options, deep brain stimulation (DBS), focused ultrasound surgery (FUS), thalamotomy, pallidotomy, and stereotactic radiosurgery (SRS) generally provide symptomatic relief for patients with PD [8-12]. These interventions aim to target specific brain regions involved in the pathophysiology of PD, thereby modulating neural activity and improving motor function. Deep brain stimulation (DBS) is the most common surgical procedure to improve motor symptoms of Parkinson's disease (PD) and reduce "off" episodes in advanced stages [8]. The two primary DBS targets, the subthalamic nucleus (STN) and the globus pallidus pars interna (GPi), provide similar motor benefits, with STN DBS allowing for reduced dopamine medication and GPi DBS causing fewer cognitive and mood side effects [8]. Evidence suggests that targeting the pedunculopontine nucleus may improve gait issues [9, 10]. Additionally, radiofrequency, radiosurgery pallidotomy, and focused ultrasound (FUS) thalamotomy are used for tremor treatment, particularly in patients unsuitable for DBS. FUS thalamotomies offer a non-invasive option but are irreversible and usually performed unilaterally, limiting their use in bilateral PD [11, 12].

Given the expanding body of research on these therapeutic modalities, it is essential to assess the scientific output and trends in this field. Through bibliometric methods, researchers can identify key publications, influential authors, and leading institutions that have shaped the current understanding and treatment of PD. This analysis also highlights the evolution of research, revealing shifts in focus, emerging trends, and the most impactful treatment approaches, such as pharmacological therapies and deep brain stimulation. Additionally, it uncovers the importance of international collaborations and networks that accelerate innovation by pooling global expertise. Ultimately, bibliometric analysis serves as a valuable tool to guide future research efforts, ensuring that emerging gaps are addressed and resources are allocated effectively to improve patient outcomes. The objectives of this bibliometric analysis were to identify research trends in Parkinson's disease treatment, analyzing the evolution of scientific output over time, and pinpointing periods of increased or decreased publication activity. The study aimed to evaluate the impact of individual researchers and institutions by assessing key metrics such as the number of publications, citations, and h-index, thereby determining their influence in the field. It also sought to explore geographical contributions by identifying the countries that contributed most significantly to research on Parkinson's treatment and highlighting international collaborations. Furthermore, the study aimed to uncover the primary focus areas in the field by analyzing keyword co-occurrence, identifying key topics such as pharmacological interventions and surgical treatments. Additionally, it evaluated collaboration networks between countries, institutions, and authors, mapping the extent and influence of these relationships. Lastly, the study aimed to assess the impact of various journals by analyzing citation data to identify the most influential sources for disseminating Parkinson's disease treatment research.

## Review

Data sources and search strategies

The inclusion criteria for the bibliometric analysis included the use of the Web of Science Core Collection (WoSCC) database and the selection of specific types of publications such as articles, review articles, proceeding papers, and early access documents. The search was based on relevant keywords related to Parkinson’s disease treatment, including "Treatment," "Therapy," "Deep Brain Stimulation," "Gamma Knife," "Focused Ultrasound Surgery," "Thalamotomy," "Pallidotomy," and "Stereotactic Radiosurgery." The exclusion criteria involved all criteria that are not mentioned in the inclusion criteria. The scientific studies were retrieved from the Web of Science Core Collection (WoSCC) database on July 12, 2024. Both the search and download were conducted on the same day to prevent any fluctuations in citation counts due to daily database updates. To achieve a thorough analysis of publication trends, data were gathered without limiting the publication year. Filters were applied to include only articles, review articles, proceeding papers, and early access documents. The search utilized the following keywords: (Treatment OR Therapy OR therapeutics OR "Deep Brain Stimulation" OR Gamma Knife OR Focused Ultrasound Surgery OR Thalamotomy OR Pallidotomy OR "Stereotactic Radiosurgery") AND ("Parkinson´s disease"). A total of 3,724 publications were downloaded in “Plain Text” format, with the record content set to “Full Record and Cited References.” After screening, no duplicates were identified. As this study used secondary data, ethical approval was not required.

Bibliometric analysis

Our bibliometric analysis advanced from a broad overview to detailed insights, covering countries/regions, authors/institutions, journals, documents/references, keywords, and research trends, despite the vast and complex nature of the data. The collected data were imported into Biblioshiny (R version 4.2.2; Institute for Statistics and Mathematics, Vienna, Austria; www.r-project.org) and VOSviewer (version 1.6.18; Centre for Science and Technology Studies, Leiden University, The Netherlands) for further analysis. Biblioshiny was primarily utilized to visualize and analyze sources, authors, and documents, offering a range of bibliometric indicators to assess the output of countries, authors, institutions, and journals [13]. Productivity was measured by the number of articles, while total citations indicated impact within the academic community, and local citations assessed impact within specific fields. These three dimensions are essential for evaluating research quality. The h-index, reflecting both productivity and impact by counting the number of papers (h) that have been cited at least h times, was also employed.

VOSviewer was used to create country/institutional collaboration plots and to map co-occurrence, co-citation, and keyword co-occurrence. The following settings were used: “create a map based on bibliographic data”, "read data from bibliographic database files”, “web of science”. Regarding the type of analysis and counting method, co-authorship and citation were performed using the full counting method. The “ignore documents with a large number of authors” option was selected and set to a maximum of 25 authors per document. Analysis was performed for the three units of analysis: authors, organizations, and countries. The minimum number of documents for author analysis was set to 10 (107 results) and the weight was based on document number. The minimum number of documents for organization analysis was set to 15 (92 results) and the weight was based on citation. The minimum number of documents for country analysis was set to 10 (45 results) and the weight was based on document citation.

Results

Of the 3,724 publications retrieved from the database, four were retracted publications and were thus not included in our analysis. The distribution from 3,720 publications was as follows: 72.52% were articles, followed by reviews (22.85%), proceeding papers (3.92%), and other types such as editorial materials, book chapters, and early access documents. The majority of these publications were written in English (97.82%), with smaller proportions in Spanish (0.64%), French (0.59%), German (0.30%), and other European and Asian languages. For our analysis, we included articles published in all languages.

Publication output and sources

**Figure 1:**
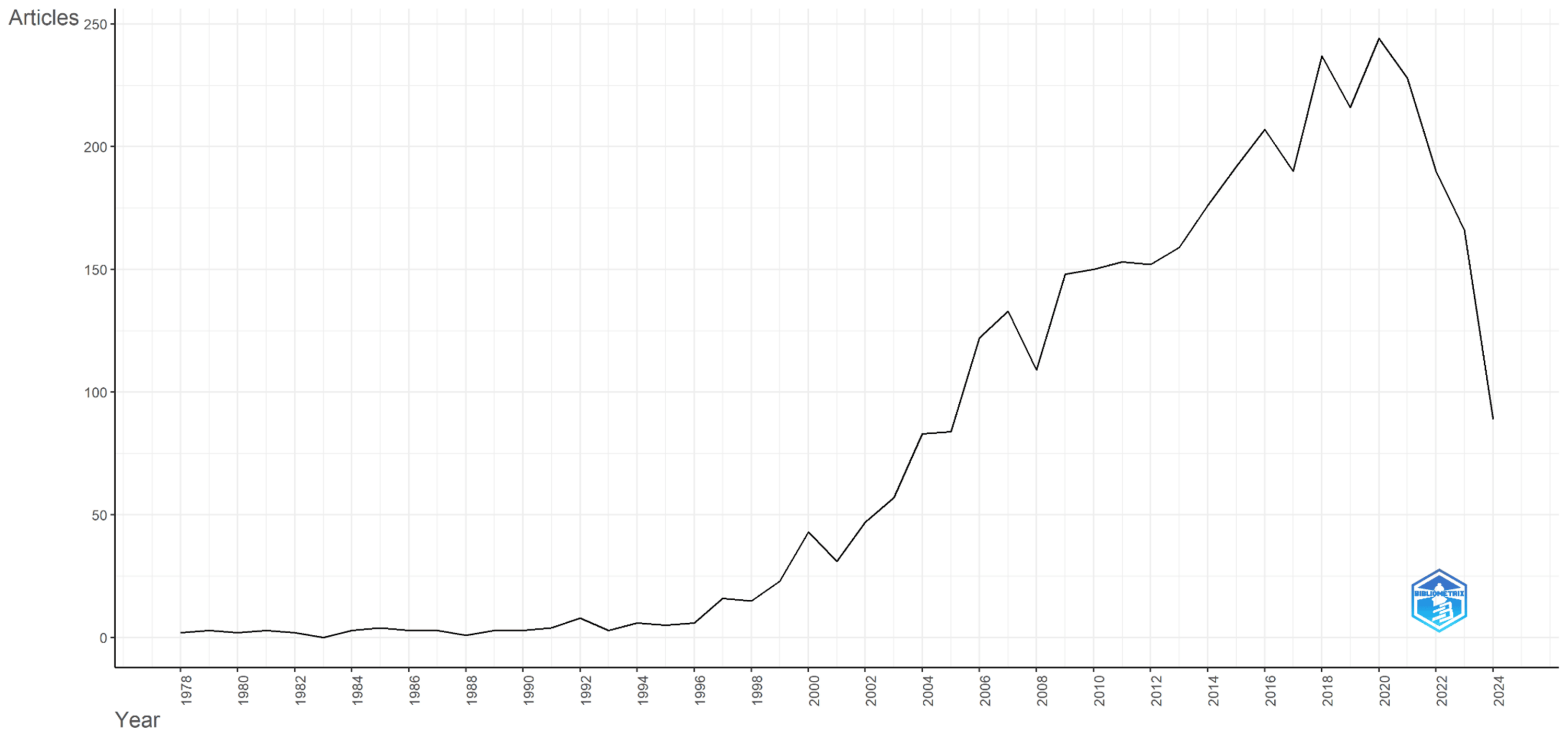
The number of scientific articles published per year from 1970.

**Figure 2:**
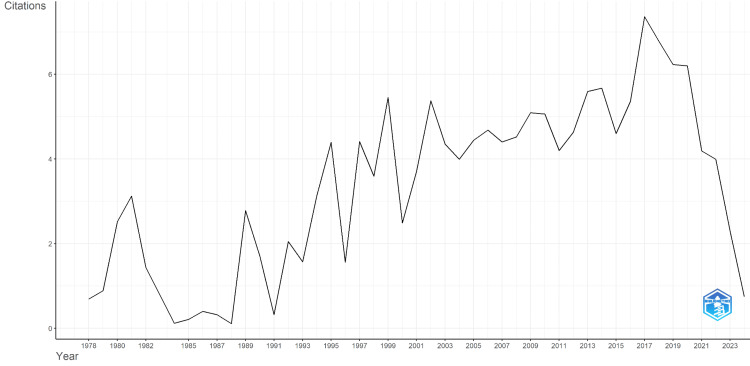
The trend for the average citations per year for published articles on Parkinson's treatment research from 1975.

The total publication trends and average citation per year were calculated and are shown in Figure 1 and Figure 2, respectively. The number of articles remained relatively low and stable, with minor fluctuations from 1978-1998. Starting around 1990, there is a noticeable increase in scientific production, with a more significant upward trend beginning around 2000. The number of articles published each year continued to rise, reaching a peak between 2011 and 2016, where it exceeded 200 articles per year. This peak suggests significant progress and breakthroughs in Parkinson's disease treatment. After 2016, there was a decline in the number of publications.

In the initial years (1975-1985), the average number of citations was low with occasional spikes, particularly around 1980 (Figure 2). From 1985 to 2000, there was a notable increase in citations, marked by peaks and troughs, indicating varying levels of interest and relevance. The period from 2000 to 2020 saw a more consistent upward trend, peaking around 2017-2018 with an average of over six citations per year. However, post-2020, there is a sharp decline in citations, dropping significantly by 2023.

Analysis of the most relevant and most cited sources in Parkinson's treatment research showed that "Neurology" was the most prolific journal with 275 articles (Table 1), followed by " Clinical Neuropharmacology" with 138 articles, and several others with over a dozen articles each. On the citation front, "Neurology" leads with 27190 citations, followed by "Archives of Neurology" and "Journal of Neurosurgery," with 17688 and 7009 citations respectively (Table 2). Other highly cited journals include "Annals of Neurology," "JAMA of Neurology," and "Journal of Biological Chemistry." This highlights the significant contributions and influence of these journals in the field.

**Table 1:** Most relevant sources for research on Parkinson's disease treatments. H-index = Hirsch index

Sources	H-Index	Articles
Neurology	411	275
Clinical Neuropharmacology	85	138
Archives of Neurology	317	131
Journal of Neurosurgery	236	113
World Neurosurgery	115	93
Annal of Neurology	331	64
JAMA Neurology	272	64
Parkinsonism & Related Disorders	122	62
Journal of Biological Chemistry	556	60
Neurosurgery	236	50

**Table 2:** Most cited sources for research on Parkinson's disease treatments. TC = total citations

Most cited Sources	TC
Neurology	27190
Archives of Neurology	17688
Journal of Neurosurgery	7009
Annals of Neurology	8733
JAMA Neurology	6020
Journal of Biological Chemistry	5518
Nature Reviews Neurology	10446
Clinical Neuropharmacology	3949
Proceedings of the National Academy of Sciences of the United States of America	6331
Parkinsonism & Related Disorders	1989

The most cited documents were identified and are indicated in Table 3 below. Leading the list is a 2017 paper by Poewe W. in Nature Reviews Disease Primers with 2822 citations, averaging 352.75 citations per year and a normalized TC of 47.95. Following are Hou YJ's 2019 Nature Reviews Neurology article with 1449 citations, Armstrong MJ's 2020 paper in JAMA with 1293 citations, and Khalil M's 2018 work in Nature Reviews Neurology with 1150 citations. The list includes a mix of high-impact papers published between 1999 and 2020 across prestigious journals such as JAMA, Nature Medicine, and Archives of Neurology.

**Table 3:** Most cited documents for research on Parkinson's disease treatments. TC = Total citations

Paper, and Journal Name	Citation	Total Citations	TC per Year	Normalized TC
POEWE W, 2017, NAT REV DIS PRIMERS	[14]	2822	352.75	47.95027723
HOU YJ, 2019, NAT REV NEUROL	[15]	1449	241.5	38.79325731
ARMSTRONG MJ, 2020, JAMA-J AM MED ASSOC	[16]	1293	258.6	41.68212446
KHALIL M, 2018, NAT REV NEUROL	[17]	1150	164.2857143	24.2180558
WEAVER FM, 2009, JAMA-J AM MED ASSOC	[18]	1095	68.4375	13.44449975
WEINTRAUB D, 2010, ARCH NEUROL-CHICAGO	[19]	998	66.53333333	13.16043956
GILL SS, 2003, NAT MED	[20]	993	45.13636364	10.38169479
CONNOLLY BS, 2014, JAMA-J AM MED ASSOC	[21]	971	88.27272727	15.55721438
LIBERATORE GT, 1999, NAT MED	[22]	904	34.76923077	6.381829343
SARKAR S, 2007, J BIOL CHEM	[23]	896	49.77777778	11.30626186

Country scientific production

**Figure 3:**
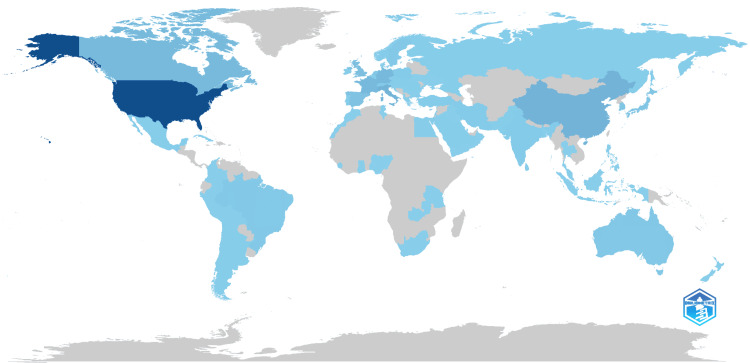
The global distribution of scientific research output related to the treatment of Parkinson's disease. Darker shades represent higher number of publications, while lighter shades represent lower number of publications.

The bibliometric analysis on Parkinson's disease showed a global distribution of scientific publications, with the United States leading in scientific production (darkest blue in Figure 3). Significant contributions also came from Canada, the UK, Germany, France, China, Japan, Australia, and Brazil (various shades of blue). In contrast, many countries in Africa, parts of Asia, South America, and Eastern Europe (shaded in gray) had lower or negligible scientific output. This map highlights geographical disparities, with the highest research concentrations in North America, Western Europe, and parts of Asia.

Analysis revealed that the United States led in Parkinson's disease research with 5479 publications, accumulating 91191 citations and averaging 65.8 citations per article (Table 4). Canada and the United Kingdom also had high impact, with Canada averaging 76.1 citations per article across 814 publications, and the United Kingdom averaging 94.1 citations per article from 613 publications. Notably, Austria, with only 81 publications, had the highest average citations per article at 191.8, indicating a significant impact. Other major contributors included France, Italy, Germany, and China, each with substantial publication counts and varying citation impacts.

**Table 4:** Country contribution to research on Parkinson's disease treatments.

Country	Total Citations	Average Article Citations	Freq
USA	91191	65.8	5479
Canada	14083	76.1	814
United Kingdom	13076	94.1	613
France	9646	63.5	856
Italy	9061	39.2	1032
Germany	7868	45.2	834
China	7271	23	1098
Austria	5754	191.8	81
Spain	4462	36	497
Japan	4278	35.9	347

**Figure 4:**
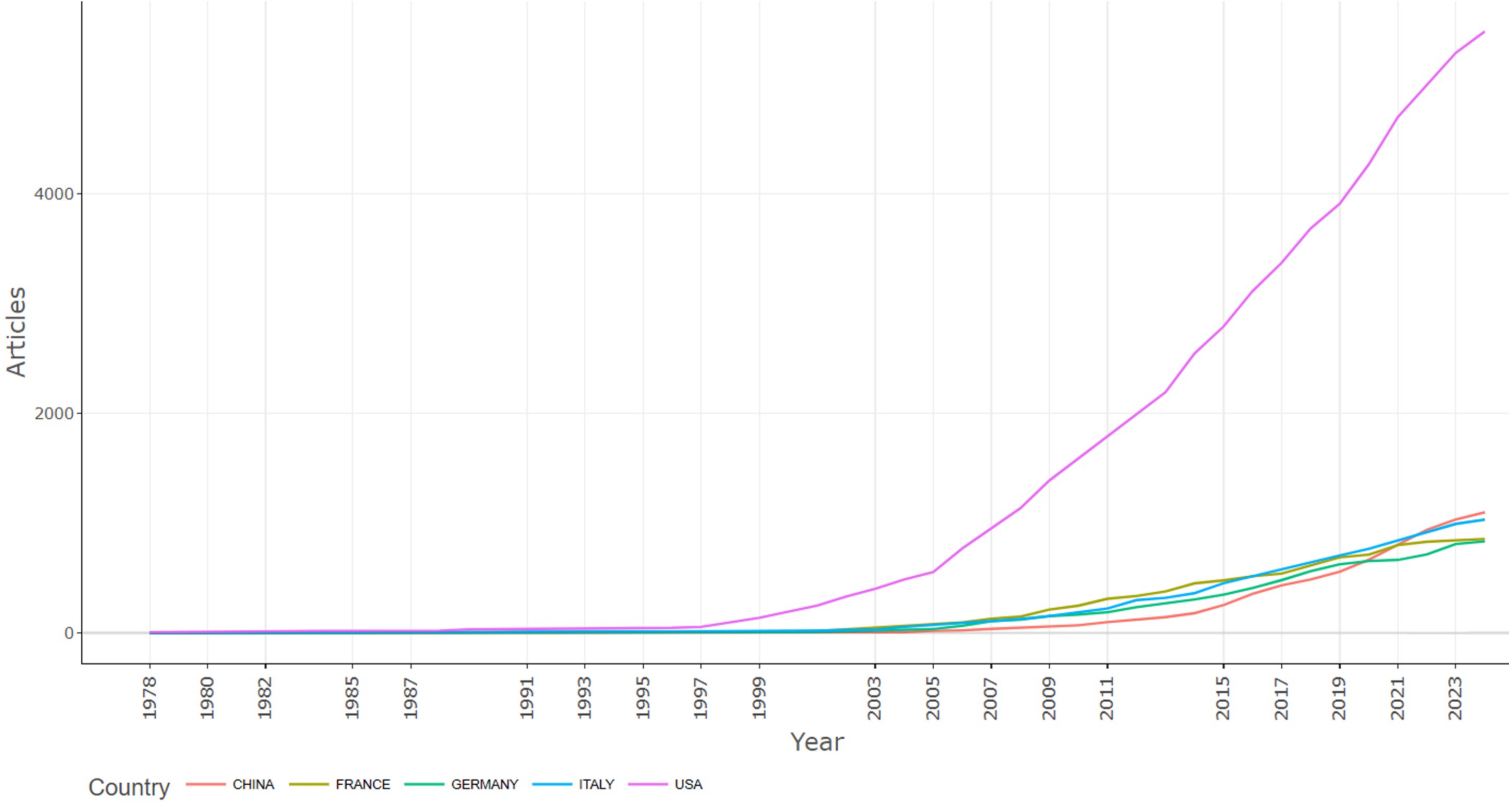
Country production over time of scientific research output related to the treatment of Parkinson's disease.

The scientific research output related to Parkinson's disease treatment was plotted over time to examine the performance of the top five countries (Figure 4). The top five countries were China, France, Germany, Italy, and the USA. China's production increased dramatically from around 2000, surpassing all others by 2023. The USA maintained a high and steadily growing output. France, Germany, and Italy exhibited similar, moderate growth patterns. The overall trend indicated a significant rise in global scientific publications, with China experiencing the most rapid increase.

**Figure 5:**
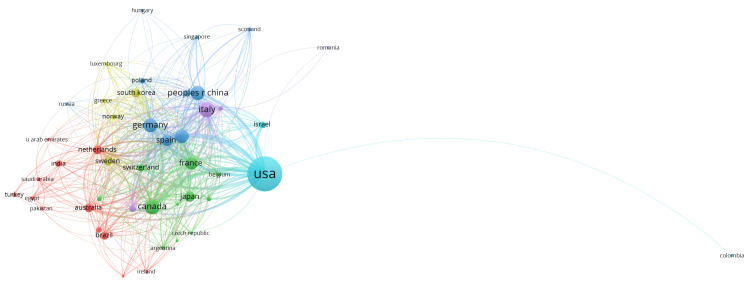
Country collaboration in scientific research related to Parkinson's disease treatment. Generated with Vosviewer. * Each node represents a country, with the size of the node indicating the volume of research output and the lines connecting nodes representing collaborative efforts between countries*.

Analysis of collaboration among countries showed that the United States (USA) was the largest and most central node, indicating its dominant role and extensive international collaborations (Figure 5). Other significant nodes included Germany, the United Kingdom, Canada, and Australia, all showing high levels of research output and collaboration. Clusters of interconnected countries highlighted regional cooperation in Europe (e.g., Germany, Italy, Spain) and Asia (e.g., China, Japan, South Korea). There were six clusters, and 488 links from 45 countries, with a total link strength of 2699. The map also showed long-distance collaborations, such as between the USA and France, China, and Australia. This visualization underscored the global nature of Parkinson's disease research and the importance of international collaboration in advancing scientific knowledge and innovation.

**Figure 6:**
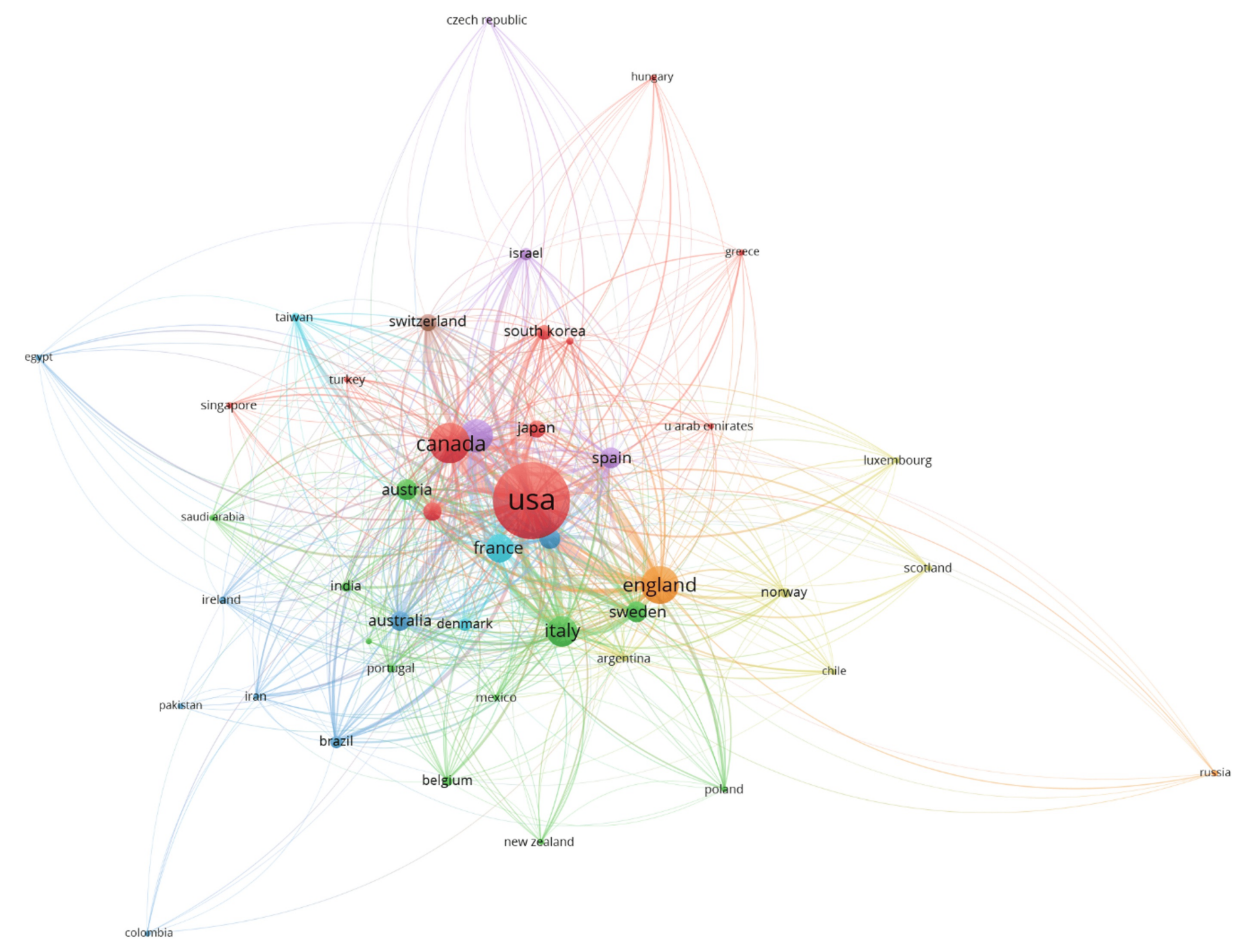
The network visualization map illustrates collaborations between countries in the field of Parkinson's disease treatment. Each node represents a country, and the links between nodes indicate citation ties*.

The network graph for citation relationships between countries based on academic publications, with weight based on citation was generated (Figure 6). There were eight clusters, and 591 links from 45 countries, with a total link strength of 12661. The USA, Canada, England, and France are prominent nodes, indicating their substantial contributions to the literature. The connecting lines (edges) between nodes depict citation links, with thicker lines representing stronger citation relationships. The color coding of the nodes and edges represents different clusters or groups of countries that frequently cite each other, suggesting regional or collaborative research trends. Notably, the USA forms the central hub, heavily interconnected with numerous countries, highlighting its significant influence and collaborative nature in global academic research. Other notable clusters include European countries like France, England, and Italy, and Asian countries such as Japan and South Korea.

Author and institutional scientific production

Lang AE, Okun MS, Lozano AM, Fernandez HH, Hauser RA, Schapira AHV, Pahwa R, Moro E, Jankovic J, and Rascol O were identified as the most relevant authors (top 10) based on article numbers (Table 5). The number of articles published by an author ranged from 27 (lowest) to 54 (highest). The University of Toronto, University of California System, Harvard University, State University of Florida, University of London, University of Pennsylvania, Washington University (WUSTL), University Health Network Toronto, Institut National de la Santé et de la Recherche médicale (INSERM), and University College London were the top 10 contributors based on article numbers (Table 6). The number of articles published by an institution ranged from 181 to 370 (Table 6).

**Table 5:** Most relevant authors for research on Parkinson's disease treatments, and their respective affiliations. H - index = Hirsch index

Authors	Affiliation	H - Index	Articles
Lang AE	University of Toronto	184	54
Okun MS	Norman Fixel Institute for Neurological Diseases	105	54
Lozano AM	University of Toronto	148	43
Fernandez HH	Cleveland Clinic	85	40
Hauser RA	University of South Florida	107	33
Schapira AHV	University College London	139	33
Pahwa R	University of Kansas Medical Center	94	32
Moro E	Grenoble Alpes University	79	31
Jankovic J	Baylor College of Medicine	194	29
Rascol O	Université Toulouse	126	27

**Table 6:** Leading institutions in Parkinson's disease research

Institutions	Articles
University of Toronto	370
University of California System	291
Harvard University	246
State University of Florida	244
University of London	235
University of Pennsylvania	207
Washington University (WUSTL)	204
University Health Network Toronto	199
Institut National de la Santé et de la Recherche médicale (INSERM)	198
University College London	181

**Figure 7:**
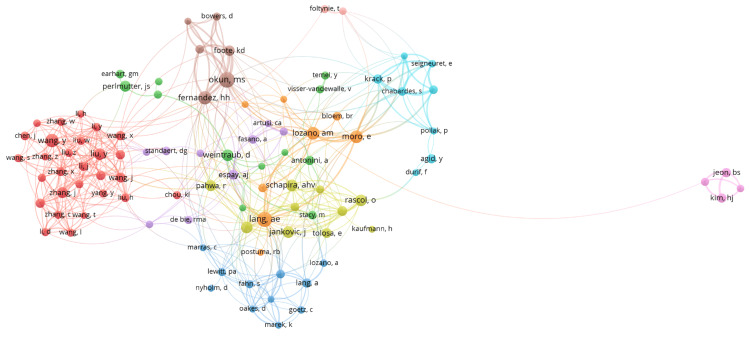
The network visualization map illustrates collaborations between researchers in the field of Parkinson's disease treatment. Each node represents an author, and the links between nodes indicate co-authorship ties*.

Author collaboration network analysis showed various clusters among researchers (Figure 7). There were 10 clusters, and 489 links from 98 countries, with a total link strength of 1147. The network features several distinct clusters, each represented by different colors, suggesting different research groups or areas of focus. For instance, the red cluster is densely connected, highlighting strong collaboration among authors like Zhang, Wang, and Liu. Other prominent clusters include a green cluster with authors like Earhart and Perlmutter, an orange cluster featuring Lang and Lozano, and a light blue cluster with Chabardes and Krack. Additionally, there is an isolated purple cluster with authors Jeon and Kim, indicating limited collaboration with other groups. Overall, the network shows a rich structure of intra-group collaborations with fewer inter-group connections.

**Figure 8:**
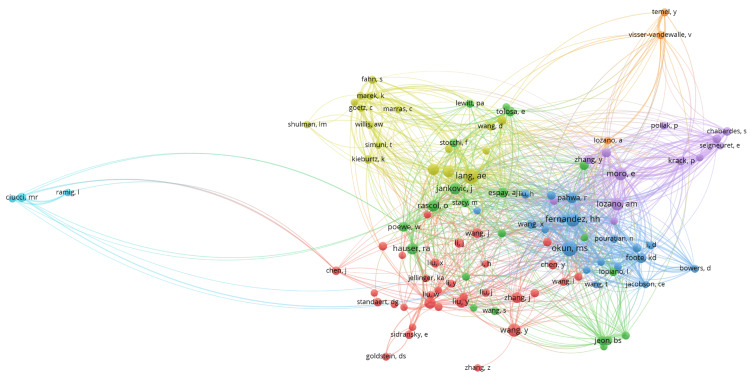
The network visualization map illustrates collaborations among authors in the field of Parkinson's disease treatment. Each node represents an author, and the links between nodes indicate citation ties*.

This citation analysis of authors showed prominent clusters of interconnected nodes, indicating groups of authors who frequently cite each other's work (Figure 8). There were seven clusters, and 1885 links from 107 countries, with a total link strength of 5216. One prominent cluster in the center-right consisted of authors like Rascol O, Poewe W, and Hauser RA, suggesting a strong collaborative or thematic linkage among them. Another distinct cluster towards the left includes authors like Ciucci MR and Ramig I, which appears more isolated compared to the central clusters. The various colors represent different clusters or communities within the citation network, highlighting the interconnectedness and collaborative nature of research among these authors.

**Figure 9:**
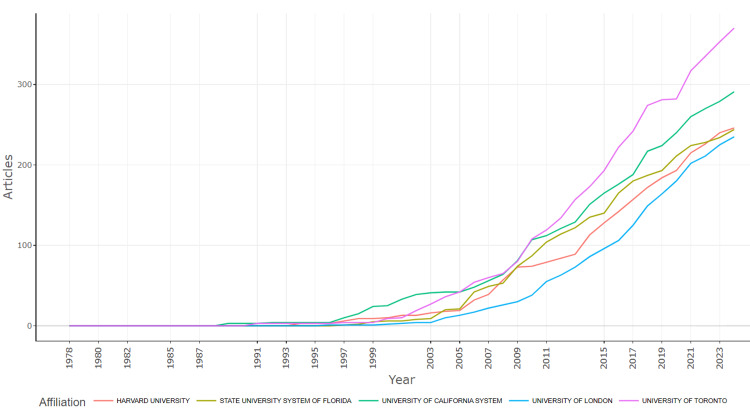
Top five institutional production over time of scientific research output related to the treatment of Parkinson's disease.

A graph illustrating the number of articles produced over time by the five top academic institutions based on the number of published articles was generated (Figure 9). Harvard University, State University System of Florida, University of California System, University of London, and University of Toronto had minimal publication output around 1989. After 1995, there was a notable increase in the number of articles produced by each institution, with a significant rise starting around the year 2000. The University of Toronto showed a steady and consistent increase, leading the output by 2023 with over 300 articles. The University of California System, the State University System of Florida, Harvard University, and the University of London also demonstrated considerable growth, each exceeding 200 articles by 2023. The University of London, although showing a significant increase, trails slightly behind the other institutions. The data highlights growth in academic productivity across these institutions over the past few decades.

**Figure 10:**
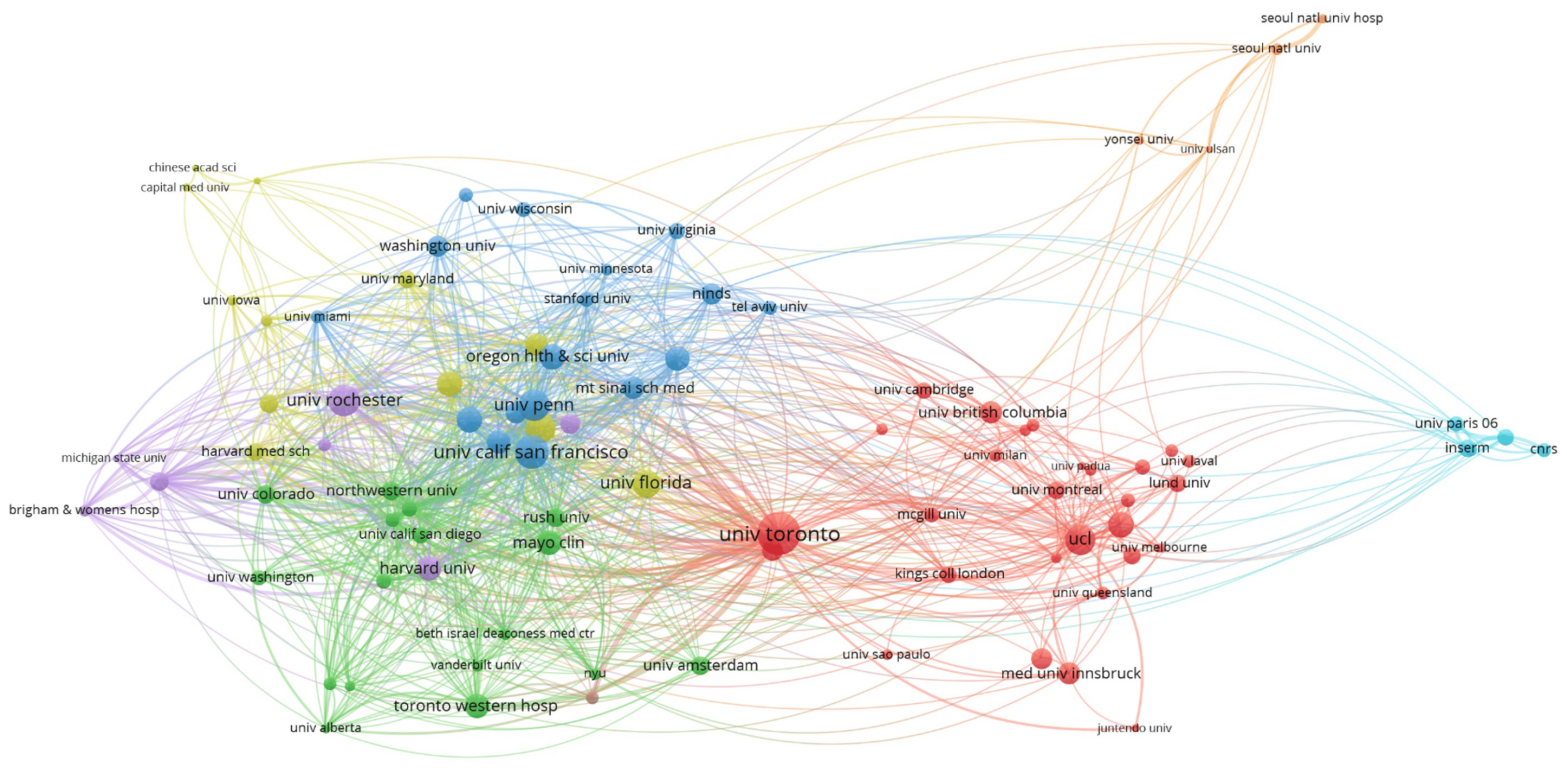
The network visualization map illustrates collaborations between institutions in the field of Parkinson's disease treatment. Each node represents an institution, and the links between nodes indicate co-authorship ties*.

Analysis for institutional collaboration among various universities was performed, with nodes representing institutions and edges indicating the strength of their collaborative relationships (Figure 10). There were eight clusters, and 1128 links from 91 countries, with a total link strength of 2154. The size of the nodes corresponds to the volume of publications with weight of analysis based on citations, with prominent institutions like the University of Pennsylvania, University of Toronto, University College London (UCL), and Seoul National University demonstrating significant collaborative networks. The graph revealed dense clusters of collaboration within regions, particularly among North American and European institutions, with substantial interconnections between these clusters and notable contributions from Asian institutions. This visualization highlights the central role of leading universities in fostering global research partnerships and the interconnected nature of contemporary academic collaborations.

**Figure 11:**
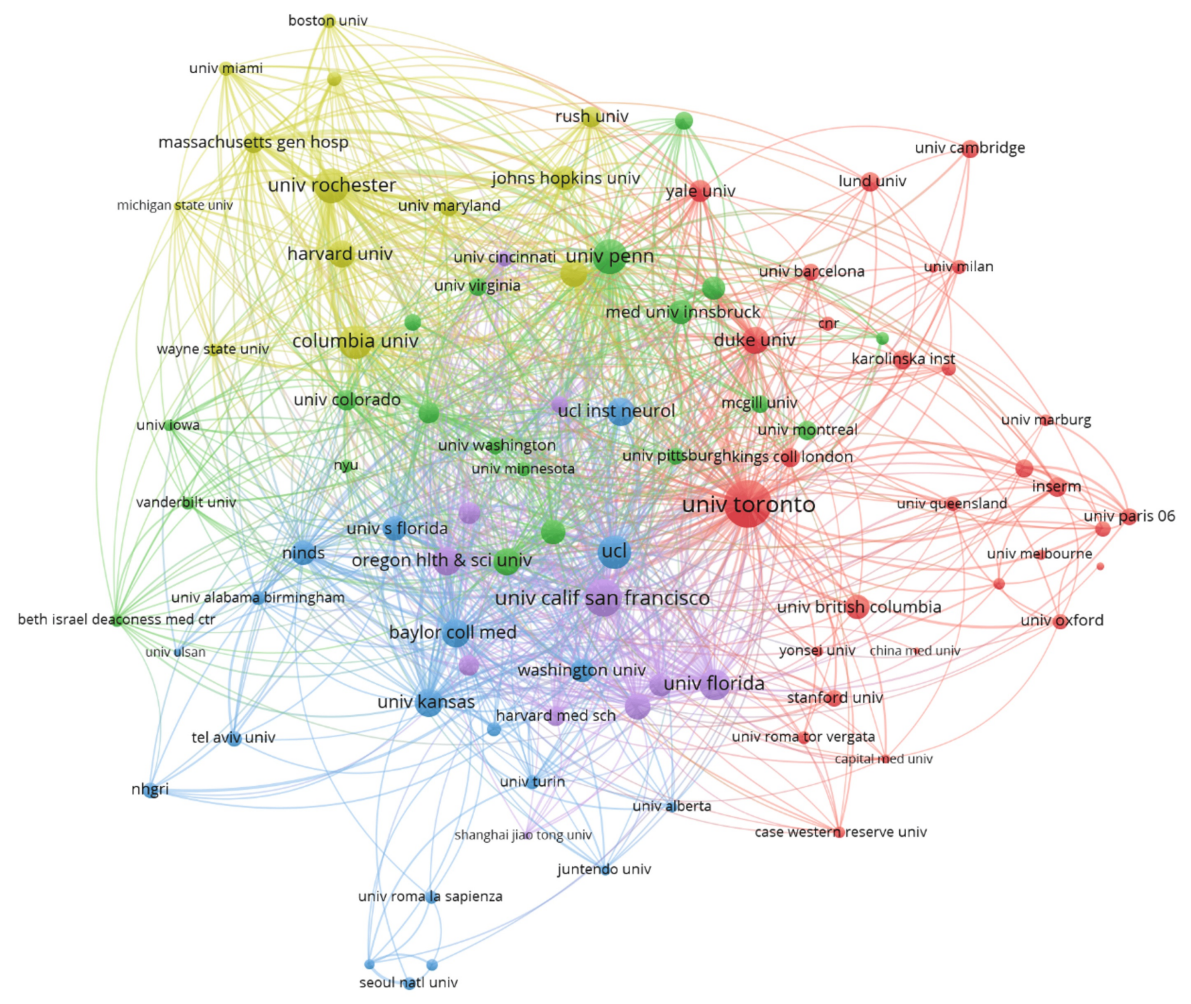
The network visualization map illustrates collaborations between institutions in the field of Parkinson's disease treatment. Each node represents an institution, and the links between nodes indicate citation ties*.

A citation analysis of various institutions revealed five interlinked clusters (Figure 11), demonstrating collaborative networks. There were five clusters, and 2707 links from 92 countries, with a total link strength of 10546. The red cluster included prominent institutions like the University of Toronto, Duke University and Yale University, indicating strong citation ties within this group. The interconnectedness of the nodes suggests a high level of collaboration and citation exchange among these institutions. Notably, major hubs such as Johns Hopkins University, Harvard University, Columbia University, and the University of California, San Francisco are central, indicating their significant influence and extensive collaborative networks in research. This highlights how key institutions serve as major contributors and connectors in the scientific community.

Discussion

Publication Output and Sources

This comprehensive bibliometric analysis of Parkinson's disease (PD) treatment research revealed significant trends and highlights influential contributions over the past several decades. The notable increase in research output from 2000 onwards, peaking around 2017-2018 underscores a period of substantial scientific progress and breakthroughs. The subsequent decline in publications after 2020 suggests a shift in research focus or potential external influences such as funding reallocations, publication delays or the global impact of the COVID-19 pandemic [24]. Despite this decline, the trend indicates a substantial increase in research activity and interest over the past few decades, followed by a recent decline that may warrant further investigation to understand the factors contributing to this decrease. Global events like the COVID-19 pandemic might have also influenced research priorities and outputs [24]. The citation trends in Parkinson's disease research highlight a dynamic field with periods of significant growth and impact. The rise in citations up to 2020 indicates a growing recognition of the importance of this field and the accumulation of significant scientific knowledge (Figure 2). The recent decline in citations calls for a closer look to understand and address potential issues, ensuring sustained progress in the fight against Parkinson's disease. Supporting and prioritizing research in this area is essential to develop new treatments and improve patient outcomes.

Higher total citations for recent articles suggest they are more influential or that there has been more research activity in recent years. The highest average citation per year by Poewe W (Table 3) suggests a significant impact within a few years of publication. Articles from 1999-2010 have a lower number of citations, which may be due to the natural ageing of research impact or differences in the volume of research over time. Recent articles got higher average citations per year, indicating that newer research is being cited more frequently in a shorter period. Higher H-index values of the journals suggest that articles published in them are consistently cited by other influential papers.

Country Scientific Production

An analysis of the collaborative networks among various countries and regions highlighted that developed Western nations, including the USA, Germany, the United Kingdom, Canada, and Austria, demonstrated dominance in Parkinson’s disease treatment research. The United States is by far the largest contributor to Parkinson's disease research with 5,479 publications. This can be attributed to its vast research system, high investments in science and health, and the presence of numerous research institutions and renowned universities. The USA has major medical centers and funding programs that encourage research in Parkinson's disease research. In recent years, China has significantly increased its investment in research and development, which is reflected in the growth of scientific publications. China is focused on expanding its role in global science, with a strong push in the fields of biotechnology and medicine. China, despite being the second-largest in scientific production, had an average of only 23 citations per article, indicating a large quantity of research but relatively low impact. Italy, with 1032 publications, is an important research center in Europe, especially in the field of neuroscience. Italy showed a substantial volume of research but relatively lower impact in terms of citations. France and Germany also demonstrated a strong presence in Parkinson's research, though with lower average citation rate compared to the US and UK. Austria produced fewer articles but they were highly cited, reflecting their significant impact in the field.

In contrast to the aforementioned developed nations, regions such as Northern South America (e.g., Colombia, which has only a collaborative network with the USA), Eastern Europe (e.g., Romania and Slovakia), and the Middle East (e.g., Lebanon and Jordan) had fewer collaborative networks. Despite the interest that some countries may have in collaborating, language barriers are recognized as obstacles to international collaboration [25]. It is encouraging to see that a few African countries, such as South Africa and Egypt, were actively engaged in collaborative clusters focused on Parkinson's disease treatment research. Their substantial academic contributions may inspire more researchers to enter this field and promote international collaboration and funding opportunities. This is particularly important because there is considerable potential for further study of Parkinson's disease on the African continent.

The quality and quantity of publications are strongly influenced by the research infrastructure and investments in the science and technology of each country. International collaborations and partnerships between institutions from different countries are common and help increase the quantity and quality of published research. Countries with fewer publications, such as those in Africa and some parts of Asia and Latin America, may face challenges related to funding, infrastructure, and training of personnel.

Author and Institutional Scientific Production

The leading authors listed in our study (Table 5) were affiliated with research institutions in developed countries. Authors such as Lang AE, Okun MS, Lozano AM, Fernandez HH, and Hauser RA made substantial impacts with their high H-indices and numerous publications, signifying the quality and influence of their research. Lang AE (H-index: 184, 54 articles) stood out as a leading figure in the field, affiliated with the University of Toronto, which also topped the institutional ranking with 370 articles. Okun MS (H-index: 105, 54 articles), associated with the Norman Fixel Institute for Neurological Diseases, and Lozano AM (H-index: 148, 43 articles) from the University of Toronto also had significant research output. Fernandez HH (H-index: 85, 40 articles) from the Cleveland Clinic and Hauser RA (H-index: 107, 33 articles) from the University of South Florida were among the top contributors to the breadth of research with their extensive publications and impact on the field.

Lang AE, Okun MS, Lozano AM, and Fernandez HH had established extensive collaboration networks. With the most substantial link strength, Lang AE and Okun MS had the highest number of publications (n=54) related to Parkinson's disease and its treatment (Table 5). Lang AE and Lozano AM belonged to the same cluster based on link strength, having co-authored publications (Figure 7). Their recent article reviewed current evidence on dopaminergic treatment options for early-stage Parkinson's disease, aiding clinicians and patients in selecting treatments and guiding counseling, prescribing, and monitoring of efficacy and safety [26]. In a collaborative study, Okun MS and Fernandez HH demonstrated that deep brain stimulation (DBS) surgery significantly improved motor symptoms in patients, regardless of the targeted brain area [27]. Lozano AM and Moro E reported that STN and GPi DBS effectively improved motor symptoms of Parkinson's disease, with sustained benefits observed at the 5 to 6-year follow-up [28]. Despite being part of the well-connected and prolific scientific community in the USA, some authors had participated in fewer collaborative research projects than their peers. Analysis showed a need for increased engagement in collaborative efforts within the field of Parkinson's disease treatment research.

The prominence and impact of research institutions significantly contribute to a country's academic standing within a specific research domain [29]. The University of Toronto led with the highest number of articles, emphasizing its pivotal role in advancing the understanding and treatment of Parkinson's (Table 6). Other top institutions included the University of California System, Harvard University, the University of London, and University College London, all significantly contributing to the field. These institutions' high article counts and rankings (Q1 or Q2) highlighted their research strength and commitment to addressing Parkinson's disease. The diversity of these institutions, from North America to Europe, underscored the collaborative and international nature of Parkinson's research. This widespread engagement across prestigious universities and research centers ensured a comprehensive approach to tackling Parkinson's disease's complexities, fostering innovation and progress in developing effective treatments.

Network analysis of institutional collaborations in Parkinson’s disease treatment research highlighted a dominance of institutions from Canada, the United Kingdom, and the United States, such as the University of Toronto, University College London (UCL), and the University of California San Francisco. This visual analysis underscored that the most active and prominently featured institutions are predominantly well-established universities from developed countries with abundant academic resources. Consequently, a noticeable disparity exists in the exchange of academic resources between developing and developed countries.

The limitations of this study include its reliance on the Web of Science Core Collection (WoSCC) database, which may not encompass all relevant publications, potentially omitting significant studies indexed in other databases. The analysis is primarily based on the quantity of publications and citations, which may not fully capture the quality or clinical impact of the research. Additionally, there is a language bias towards English, which could lead to the underrepresentation of research published in other languages. The study also notes a decline in publication output after 2020, possibly influenced by external factors such as the COVID-19 pandemic, but this is not explored in depth. Finally, while the study highlights contributions from leading countries and institutions, it also reveals regional disparities, with less representation from developing regions, indicating a need for more inclusive global research efforts.

## Conclusions

This bibliometric analysis elucidated the trends and impacts of scientific contributions, emphasizing the prolific output from leading countries and institutions in relation to the treatment of Parkinson's disease. Take-home messages for the conclusion of our study are as follows: (1) this study found a substantial increase in Parkinson's disease (PD) research output from 2000 onwards, peaking around 2017-2018, (2) noted a decline in publication output post-2020, (3) the United States had the highest research output, followed by significant contributions from countries like China, Canada, and the United Kingdom, (4) international collaborations played a vital role in advancing PD research, (5) key researchers in the field were Lang AE, Okun MS, and Lozano AM, (6) and established institutions like the University of Toronto, Johns Hopkins University and Harvard University made substantial contributions to the field, emphasizing the role of leading academic centers in driving PD research. This study highlights the need for sustained research support and the inclusion of developing countries in collaborative works. Ensuring continued progress in PD research requires addressing recent declines in publication output and supporting new research initiatives. Sustained funding and international collaboration are crucial for developing more effective treatments and improving patient outcomes.
